# Cortical atrophy mediates the accumulating effects of vascular risk factors on cognitive decline in the Alzheimer’s disease spectrum

**DOI:** 10.18632/aging.103573

**Published:** 2020-07-29

**Authors:** Qing Wang, Cancan He, Yao Zhu, Qianqian Zhang, Zhijun Zhang, Chunming Xie

**Affiliations:** 1 Department of Neurology, Affiliated ZhongDa Hospital, School of Medicine, Southeast University, Nanjing 210009, Jiangsu, China; 2 Neuropsychiatric Institute, Affiliated Southeast University, Nanjing 210009, Jiangsu, China

**Keywords:** Alzheimer's disease, vascular risk factors, cortical atrophy, MRI, mediation analysis

## Abstract

There are increasing concerns regarding the association of vascular risk factors (VRFs) and cognitive decline in the Alzheimer's disease (AD) spectrum. Currently, we investigated whether the accumulating effects of VRFs influenced gray matter volumes and subsequently led to cognitive decline in the AD spectrum. Mediation analysis was used to explore the association among VRFs, cortical atrophy, and cognition in the AD spectrum. 123 AD spectrum were recruited and VRF scores were constructed. Multivariate linear regression analysis revealed that higher VRF scores were correlated with lower Mini-Mental State Examination scores and higher Alzheimer's Disease Assessment Scale-Cognitive Subscale scores, indicating higher VRF scores lead to severer cognitive decline in the AD spectrum. In addition, subjects with higher VRF scores suffered severe cortical atrophy, especially in medial prefrontal cortex and medial temporal lobe. More importantly, common circuits of VRFs- and cognitive decline associated with gray matter atrophy were identified. Further, using mediation analysis, we demonstrated that cortical atrophy regions significantly mediated the relationship between VRF scores and cognitive decline in the AD spectrum. These findings highlight the importance of accumulating risk in the vascular contribution to AD spectrum, and targeting VRFs may provide new strategies for the therapeutic and prevention of AD.

## INTRODUCTION

R2-1Alzheimer’s disease (AD), the leading cause of neurodegenerative dementia associated with aging, affects over 50 million individuals worldwide and is predicted to increase to 152 million individuals affected by 2050 [1]. Currently, there are no effective treatments to stop the progression of AD [2]. Therefore, reducing the risk of developing AD has increased the importance of early prevention of AD. Currently, there are increasing concerns regarding the association of vascular risk factors (VRFs) and cognitive decline in the AD spectrum since the vascular hypothesis of AD was first proposed in 1993 [3–8]. Recent reports indicated that VRFs, such as diabetes, hypertension, and current smoking, have been associated with increased risk of AD [9, 10]. It was estimated that one third of all AD cases worldwide might be attributable to potentially modifiable risk factors [11], while a 10%-25% reduction in all diabetes, midlife hypertension, mid-life obesity, smoking, depression, low educational attainment and physical inactivity risk factors could potentially prevent as many as 1.1-3.0 million cases worldwide [12]. Recent evidence from systematic review and meta-analyses suggests that antihypertensive use may lower the incidence of dementia and AD [1], and from randomized controlled trials suggest that rosiglitazone may reduce the risk of AD in patients with diabetes [13]. So public health interventions targeted at vascular risk factors will probably achieve the greatest reduction in the prevalence of AD. Recent studies showed that a cumulative number of VRFs were significantly associated with elevated brain amyloid in midlife or healthy aging [5, 14], which is a core feature of AD pathology [11, 15]. Neuroimaging studies have also found that VRFs were associated with structural disturbances in the brain, which were significantly associated with declined memory and executive function in late life [3, 16, 17], suggesting that VRFs could accelerate brain structural aging in midlife and that the vascular burden contributes to the progression of cognitive impairment to dementia. For example, structural atrophy in the posterior cingulate cortex (PCC), middle temporal structures, and entorhinal cortices has been observed in type 2 diabetes mellitus patients [18, 19]. Hypertension could increase the risk of dementia and contribute to the atrophy of the prefrontal-temporal cortex and hippocampus, which are involved in cognitive and executive function [20–22]. Decreased gray matter volumes (GMVs) were also found in the insula, parahippocampus, and amygdala in smokers compared to nonsmokers [23, 24]. Furthermore, increasing evidence has identified an association between obesity and decreased functional connectivity within networks that comprise the medial prefrontal cortex and default mode network (DMN) in healthy adults [25], while a higher educational level could decrease the risk of dementia compared with those with fewer years of formal education [4]. In addition, depression, as a severe form of psychological distress, has been associated with subcortical and hippocampal neuronal loss [26] and accompanies cognitive impairment in old adults [27]. Recently, the aggregating effects of VRFs on cerebrovascular changes have attracted more attention in AD and mild cognitive impairment (MCI) patients [28, 29]. Preliminary findings have shown that the cumulative effects of VRFs significantly enhance the cortical thinness in MCI patients and accelerated the disruption of GMVs in community-populations across middle and older age [30]. Taken together, these findings directly support the vascular hypothesis of AD and indicate the VRFs may speed up disturbances of brain structural and functional integration in the AD spectrum.

Although substantial evidence indicates a single VRF contributes to AD pathophysiology and dementia, there have been limited data to date examining the summative effects of VRFs that affect cognitive performance in the AD spectrum. Currently, we hypothesize that the VRFs burden accelerates brain structure aging in the AD spectrum and that cortical atrophy can mediate the effects of VRFs on cognitive decline. Therefore, the purpose of this study was to investigate whether the accumulating effects of VRFs contribute to gray matter atrophy and subsequently lead to cognitive decline as well as to examine the mediation effect of cortical atrophy that links the VRFs and cognitive decline in the AD spectrum.

## RESULTS

### Participant characteristics

The demographic information and clinical evaluations are shown in Table 1. No significant differences were identified for gender and age among the four groups. Notably, the apolipoprotein E (APOE) ε4 carrying status and Mini-Mental State Examination (MMSE) and Alzheimer's Disease Assessment Scale-Cognitive Subscale (ADAS-Cog) scores were significantly different (Table 1). Post-hoc analysis further indicated the source of the differences (Bonferroni correction, p<0.05/3=0.017) and identified more APOEε4 carriers and more severe cognitive impairment (lower MMSE and higher ADAS-Cog scores) in the AD group than in the other three groups. In addition, we also found that there was significantly increase of the VRF scores in the AD spectrum.

**Table 1:** Participant characteristics.

**Category**	**CN (n=69)**	**EMCI (n=52)**	**LMCI (n=41)**	**AD (n=30)**	**p value**
Age	73.6±5.9	71.2±6.7	71.5±8.1	73.1±6.8	0.462*
Gender(F/M)	40/29	31/21	17/24	15/15	0.312^†^
APOE (ε4≥1 allele)	21	26^a^	17^d^	22^c,^	0.001^†^
MMSE	28.8±1.3	28.2±1.8^e^	27.7±1.6^b,d,^	22.5±2.5^c^	<0.001*
ADAS-Cog	9.1±4.0	12.6±5.4^a,e^	16.9±6.7^b,d^	34.4±10.5^c^	<0.001*
VRF scores	1.1±1.1	1.4±1.2 ^e^	1.4±1.5^d^	2.1±1.7^c^	0.004*

Note: *, *P* values were obtained using one-way ANOVA; ^†^, *P* values were obtained by Chi-square test; unless otherwise indicated, data are presented as mean± standard deviation. Post-hoc analysis was used Bonferroni correction method; a represents statistical difference between the CN group and the EMCI group; b represents statistical difference between the CN group and the LMCI group; c represents statistical difference between the CN group and the AD group; d represents statistical difference between the AD group and the LMCI group; e represents statistical difference between the AD group and the EMCI group. **Abbreviations**: CN, cognitively normal; EMCI, early mild cognitive impairment; LMCI, late mild cognitive impairment; AD, Alzheimer’s disease; F/M, female/male; APOE, apolipoprotein E; MMSE, mini-mental state examination; ADAS-Cog, Alzheimer’s disease assessment scale-Cognitive section; VRF, vascular risk factor.

### Group-level comparison of VRFs and behavioral significance

**Figure 1:**
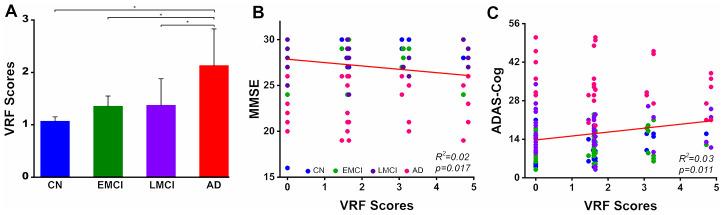
**Group-level comparison of VRF scores in the AD spectrum population and its behavioral significance.** A: The results illustrated that there was a significant increase in the VRF scores in the AD spectrum (F_(3, 188)_= 4.53, p=0.004). B and C: VRF scores were significantly correlated with cognitive impairment severity, which was measured by the MMSE and ADAS-Cog scores, after controlling for the effects of age, gender and gray matter volumes. This finding directly indicates that a higher VRF scores is associated with a greater severity of cognitive impairment. Abbreviations: VRF, vascular risk factor; CN, cognitively normal; EMCI, early mild cognitive impairment; LMCI, late mild cognitive impairment; AD, Alzheimer’s disease; MMSE, Mini-Mental State Examination; ADAS-Cog, Alzheimer's Disease Assessment Scale-Cognitive Subscale; VRF, vascular risk factor.

As shown in Figure 1, we observed that the VRF scores were significantly increased with disease severity in the AD spectrum. Importantly, the VRF scores were negatively correlated with the MMSE scores and positively correlated with the ADAS-Cog scores, after controlling for the effects of age, gender and GMV in the AD spectrum.

### Effects of VRFs on GMV across all subjects

**Figure 2:**
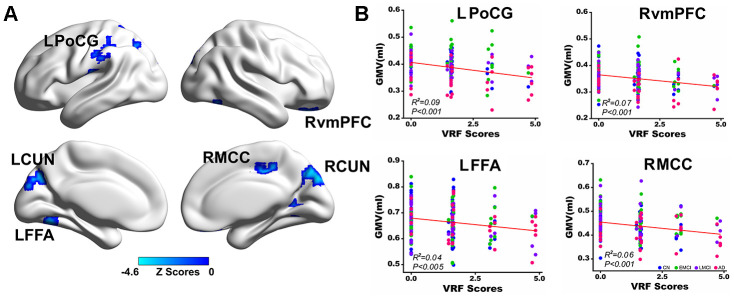
**Multivariate regression analysis indicates the effects of VRFs on gray matter volume across all subjects.** (**A**) Brain regions representing the significant effects of VRFs on GMV after controlling for the effects of covariates, including age, gender, APOEε4 genotype, and group. The blue color indicates a negative correlation between VRF scores and GMV. The color bar is presented with z scores. (**B**) Representative illustration of the significant effects of VRFs on regions of the LPoCG, LFFA, RvmPFC and RMCC. Abbreviations: VRFs, vascular risk factors; LPoCG, left postcentral gyrus; CUN, cuneus; LFFA, left fusiform face area; RvmPFC, right ventromedial prefrontal cortex; RMCC, right middle cingulate cortex; GMV, gray matter volume; CN, cognitively normal; EMCI, early mild cognitive impairment; LMCI, late mild cognitive impairment; AD, Alzheimer’s disease; APOE, apolipoprotein E.

Voxel-wise multivariate linear regression analysis identified the neural basis of VRFs on GMV across all subjects. Briefly, the VRF scores were negatively correlated with the GMV in the left postcentral gyrus (LPoCG), left cuneus (LCUN), and left fusiform face area (LFFA) and right ventromedial prefrontal cortex (RvmPFC), right middle cingulate cortex (RMCC), and right cuneus (RCUN), as shown in Figure 2A. Numerical representations of the significant effects of VRFs on the regions of the LPoCG, LFFA, RvmPFC, and RMCC are also shown in Figure 2B. In addition, the neural effects of a single VRF on GMV were also identified and described in Supplementary Figure 1.

### Group-level comparison of GMV and behavioral significance

Similarly, analysis of the voxel-wise, group-level significant difference of GMV and a post-hoc analysis were performed to detect the source of the difference in the AD spectrum. We also identified the atrophy pattern in the early mild cognitive impairment (EMCI), late mild cognitive impairment (LMCI) and AD patients compared to the cognitively normal (CN) subjects. Moreover, the neural substrates of the MMSE and ADAS-Cog on the whole-brain GMV were identified in the AD spectrum, and the detailed information is illustrated in Supplementary Figures 2–4.

### Common circuits of the VRFs and cognitive performance on GMV across all subjects

**Figure 3:**
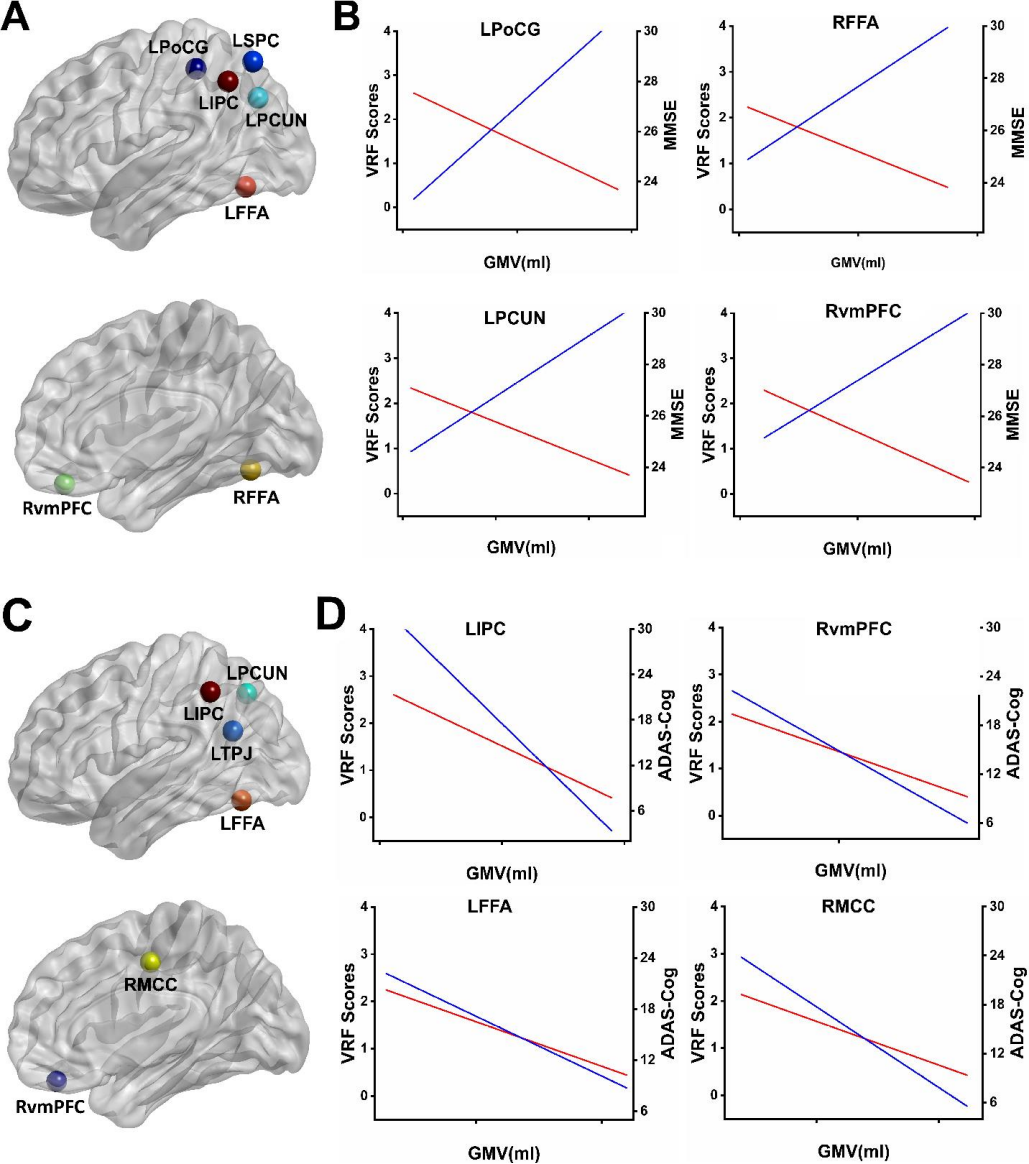
**Overlapping regions from the effects of VRFs on GMV and the correlates of cognitive performance influenced by GMV.** (**A**) Mapping the overlapping regions from the accumulating effects of VRFs on GMV and the neural correlates of the MMSE on GMV in the AD spectrum. (**B**) Representative illustration of the relationship among the VRF scores, GMV, and MMSE scores in the overlapped regions. The results indicate that higher VRF scores are associated with more GM atrophy (red lines) and a lower MMSE performance (blue lines). (**C**) Mapping the overlapped regions from the accumulating effects of VRFs on GMV and the neural correlates of the ADAS-Cog on GMV in the AD spectrum. (**D**) Representative illustration of the relationship among the VRF scores, GMV, and ADAS-Cog scores in the overlapped regions. The results indicate that higher VRF scores are associated with more GM atrophy (red lines) and a higher ADAS-Cog performance (blue lines). Abbreviations: LSPC, left superior parietal cortex; LPoCG, left postcentral gyrus; LIPC, left inferior parietal cortex; LPCUN, left precuneus; LFFA, left fusiform face area; RvmPFC, right ventromedial prefrontal cortex; RFFA, right fusiform face area; LTPJ, left temporoparietal junction; RMCC, right middle cingulate cortex; MMSE, Mini-Mental State Examination; ADAS-Cog, Alzheimer's Disease Assessment Scale-Cognitive Subscale; GMV, gray matter volume; VRF scores, vascular risk factor scores.

As shown in Figure 3A, the common regions involved in the neural basis of the MMSE and the accumulating effects of VRFs on the whole brain GMV were identified. These regions included the left superior parietal cortex (LSPC), left postcentral gyrus (LPoCG), left inferior parietal cortex (LIPC), left precuneus (LPCUN), LFFA, RvmPFC, and right fusiform face area (RFFA). Interestingly, we also found the common regions involved in the neural structures of the ADAS-cog and the accumulating effects of VRFs on the whole brain GMV in the AD spectrum, including the LIPC, LPCUN, left temporoparietal junction (LTPJ), LFFA, RvmPFC, and RMCC, as shown in Figure 3C. In addition, representative illustrations of the relationship among the VRFs, GMV, and MMSE scores or ADAS-Cog scores in the overlapped regions are presented in Figure 3B and Figure 3D.

### Mediating effects of cortical atrophy on the relationship between VRFs and cognitive performance

**Figure 4:**
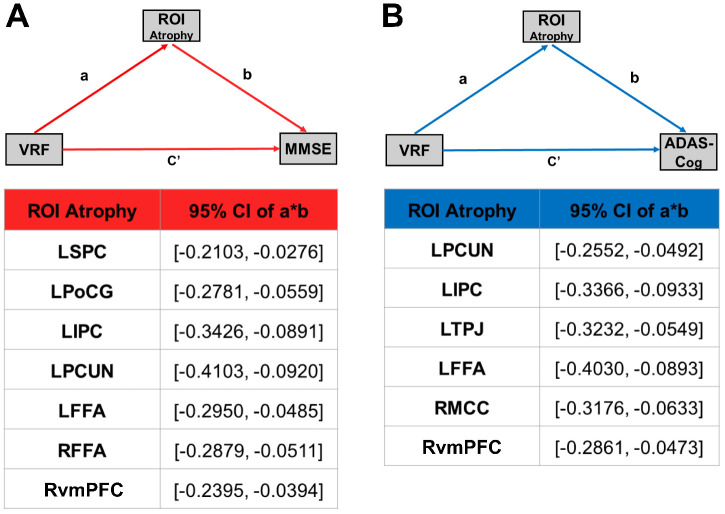
**Cortical atrophy mediates the association between VRFs and cognitive function in the AD spectrum population.** (**A**) Mediation effect of cortical atrophy linking the VRFs and MMSE in AD spectrum population. (**B**) Mediation effect of cortical atrophy linking the VRFs and ADAS-Cog in the AD spectrum population. All of the mediation effects and significance are computed by bootstrap sampling with 10,000 iterations. Effect sizes and 95% confidence intervals are displayed for each ROI. Abbreviations: LSPC, left superior parietal cortex; LPoCG, left postcentral gyrus; LIPC, left inferior parietal cortex; LPCUN, left precuneus; LFFA, left fusiform face area; RvmPFC, right ventromedial prefrontal cortex; RFFA, right fusiform face area; LTPJ, left temporoparietal junction; RMCC, right middle cingulate cortex; MMSE, Mini-Mental State Examination; ADAS-Cog, Alzheimer's Disease Assessment Scale-Cognitive Subscale; GMV, gray matter volume; VRF scores, vascular risk factor scores; ROI, region of interest; a, slope of VRF→ atrophy; b, slope of atrophy→ MMSE or ADAS-Cog; c^'^, slope of VRF→ MMSE or ADAS-Cog; a*b, the strength of the mediation pathway.

The simple mediator model of the effect of VRFs on cognitive function was employed, and cortical atrophy regions were entered into the model as candidate mediators. We used the overlapped brain areas previously described as region of interest (ROI) for the mediation analysis. Cortical atrophy was shown to partly mediate the effect of VRFs on cognitive performance. Given this finding, the 95% CI for the path a*b did not cross zero, and the indirect effect of VRFs on cognitive performance through the ROI was considered significant (Figure 4).

### Hypothesis that the neural mechanism of VRFs affects GMV in the AD spectrum

Given the accumulating effects of VRFs and individual VRFs on accelerating cortical atrophy and integrating the findings of previous studies, a system model underlying the neurobiological mechanism was proposed and might, at least in part, reflect the relationship among VRFs, cortical atrophy, and cognitive decline in the AD spectrum, as shown in Supplementary Figure 5. This model indicates that a single VRF acts on the cognitive normal brain, followed by a chronic neuroinflammatory reaction in the brain. Subsequently, chronic neuroinflammation continually dysregulates amyloid precursor protein processing, thus promoting β-amyloid (Aβ) plaque formation and the hyperphosphorylation of tau protein (two key features of AD pathology) in the cerebral cortex. This increased burden on the cerebral cortex may trigger downstream cellular and molecular events associated with AD, leading to inefficient synaptic communication and, eventually, accelerating brain atrophy. With the accumulation and propagation of chronic neuroinflammatory reactions in the brain, the decreased cognitive function of individuals also progresses from a mild cognitive impairment to a complete loss of the ability of to live and even to dementia.

## DISCUSSION

We demonstrated the accumulating effects and individualized contributions of VRFs to the cortical atrophy and, importantly, the cortical atrophy mediating the relationship between VRFs and cognitive performance in the AD spectrum. These findings strongly suggest that the VRFs burden may exacerbate subtle cognitive decline associated with cortical atrophy and indicate that the increased risk for cognitive decline in individuals with elevated VRFs may be driven by cortical atrophy [3]. Those indicate that identifying people with high VRF scores and intervening against these risk factors might be the key to prevent the occurrence of AD. Previous data from several retrospective studies have found declining dementia prevalence or incidence rates in specific population cohorts since the 1970s [31–34], especially, in those studies with the large improvements in educational attainment (including higher rates of graduation from high school and college attendance) [32–34]. Recent evidences have shown that antihypertensive, rosiglitazone and formal education are beneficial for reducing an individual’s risk for cognitive decline and dementia [1, 13, 35]. Other studies have also noted that substantial improvements in management of VRFs such as smoking and obesity, could decline the prevalence or incidence of dementia [36, 37]. Thus, targeting VRFs may provide a novel disease-modifying strategy to prevent or delay the progression of AD.

Although increasing evidence has indicated that VRFs are associated with cognitive impairment [38], most studies tend to focus on the VRFs individually, while multiple VRFs often coexist in reality. Currently, we investigated how the six common VRFs jointly affected gray matter atrophy and cognitive decline in the AD spectrum; we identified that the accumulating effects of six VRFs were associated with cognitive decline and might occur via accelerating the cortical atrophy, particularly in the prefrontal cortex and parietal-temporal system. Previous studies have reported that exposure to VRFs could accelerate structural brain aging, including hippocampal and temporal lobe atrophy, and cognitive decline in midlife, even in the absence of cerebrovascular disease [39]. Structural changes in these brain regions closely related to AD were also identified in nondemented adults with VRFs [40, 41]. Evidence has shown that hypertension, diabetes and obesity were independently and negatively associated with overlapping gray matter regions, including the posterior cingulate cortex, which overlapped with regions that are known to show atrophy in AD [42]. The negative associations of VRFs with spatial memory were associated with mediation through differences in posterior cingulate cortex volume [42]. Several studies on the cerebral structure of patients with diabetes have evidenced increased cortical and subcortical atrophy, which were associated with impaired cognitive performance [43, 44]. Recent study has also shown that depression was associated with progressive atrophy of frontal cortex and anterior cingulate cortex and contributed to more rapid conversion to dementia in MCI [45]. Moreover, increased hypertension was strongly associated with elevated Aβ deposition in healthy aging with at least 1 APOE ε4 allele [14] and AD patients [46] and, together with obesity, could moderate the relationship between Aβ deposition and cognitive decline even in midlife [47]. These findings indicate that VRFs may impact brain structural atrophy before a disease process is evident and facilitate the process of AD through an amyloid-dependent pathway. More importantly, we also identified the individualized contribution of each VRF to the cortical atrophy in the AD spectrum and indicated that distinct brain structural atrophy patterns in different stages of the AD spectrum are attributed to specific VRFs. These findings provide direct evidence to support the view that specific neural systems are differently vulnerable to the VRFs burden in this disease. From this point, our results extended previous studies that a higher VRFs burden was associated with an increased rate of progression of global brain atrophy, particularly in the medial temporal lobe, hippocampal atrophy, and cognitive decline [3, 48, 49].

It is intriguing that common brain regions involved in the VRFs and cognitive performance were identified in the AD spectrum, which are primarily located in the vmPFC and posterior parietal-temporal cortex. Accumulating evidence has frequently indicated that these brain regions were closely associated with abnormal Aβ deposition in AD patients [50], which indicates that structural overlap between VRF-impacted regions and AD-related structural changes within parietal-temporal and frontal areas may lead to an enhanced vulnerability of these regions in individuals with both conditions. More importantly, we identified that cortical atrophy in these regions could mediate the potential effects of VRFs on the cognitive decline in the AD spectrum. Previous studies have reported that these regions, as a distinctive subsystem of the DMN, present with a unique characteristic of functional-anatomic connectivity, cognitive associations, and responses to AD pathophysiology [51, 52]. Furthermore, as proposed by the cascading network failure in the AD spectrum, the failure initially starts in the posterior DMN, particularly in the posterior parietal-temporal cortex, and then transfers the processing burden to other subsystems of the DMN [51, 53]. Importantly, these overlapped regions, as the core components of the posterior DMN, were preferentially targeted by the Aβ deposition before measurable amyloid plaques [51]. These findings suggest that VRFs may synergistically act with AD pathology on cognitive decline and jointly accelerate the progression to AD.

The exact cause of high VRFs accelerating cognitive decline in adults is unknown and will require further investigation. As proposed by the two-hit vascular hypothesis, VRFs initially lead to vascular dysfunction, reduce the cerebral blood flood, disrupt the oxygenic supply and nutrients to brain tissues, and subsequently contribute to brain atrophy [54]. Moreover, VRF-related vascular dysfunction directly leads to increased productions and decreased clearance of Aβ. Multiple molecular mechanisms, such as oxidative stress, altered endothelial function, inflammation, impaired endothelial progenitor cell function, increased brain blood barrier permeability, and less clearance of beta-amyloid were involved in the process of higher risk factors promoting cognitive impairment and dementia [55]. Also, increasing insulin resistance, hyperinsulinemia and endothelial dysfunction in diabetes were associated with more hippocampal and amygdala neuronal loss and atrophy, which ultimately led to memory decline [43, 56]. In addition, it has been suggested that changes in the serotonergic system driven by neurodegeneration in adjacent cholinergic system may induce depression and accelerate cognitive impairment [57]. These processes significantly increase the risk of AD onset and progression [58]. Another hypothesis is that VRFs may induce a chronic neuroinflammation reaction of the brain, which subsequently contributes to brain structural atrophy by releasing immune mediators [59]. Accumulating evidence has suggested that chronic neuroinflammation is increasingly emerging as an important pathological factor in the development and progression of AD [60–66]. According to this theory, it is apparent that abnormal Aβ deposition can activate microglia and astrocytes, trigger an innate immune response and subsequently release inflammatory mediators, which contribute to disease development and progression. Based on these findings, we proposed a system model centered on the neuroinflammation-related mechanisms of how VRFs contribute to brain structural changes and, ultimately, result in AD. In this model, the VRFs jointly or individually affect the neurovascular unit, are likely to interfere with immunological processes of the brain, and further promote cortical atrophy and cognitive impairment. The modulation of these VRFs and targeting the immune mechanisms could provide new insights for future therapeutic or preventive strategies for AD.

There were several limitations in our study. First, our study was a cross-sectional study, and it was not possible to observe the longitudinal cognitive changes in each group. Second, previous research has shown that higher levels of VRFs were associated with poorer brain health among white matter macrostructure and microstructure damage [67], currently, we did not strictly control the effects of VRFs on the white matter changes in the AD spectrum. Third, other factors, such as hyperlipidemia, might contribute to AD pathophysiology and should be investigated in the future study. In addition, a small sample size was recruited in the current study, which may limit the generalizability of these findings. To overcome these limitations, a community-based, clinical cohort project should be performed and more subjects recruited to validate our results in follow-up studies.

The present study demonstrates the accumulating effects of VRFs on cortical atrophy and cognitive decline and shows that cortical atrophy can mediate the relationship between VRFs and cognitive performance in the AD spectrum. These findings expand our understanding of how VRFs affect brain structure changes and subsequently lead to cognitive decline and highlight that targeting VRFs may provide new strategies for the early detection, treatment and prevention of AD.

## MATERIALS AND METHODS

### The Alzheimer’s disease Neuroimaging Initiative data set

The data used in this article were obtained from the public Alzheimer’s disease Neuroimaging Initiative (ADNI) database (https://ida.loni.usc.edu). The ADNI was launched by the National Institute on Aging, the National Institute of Biomedical Imaging and Bioengineering, the Food and Drug Administration, private pharmaceutical companies and nonprofit organizations in 2003. The goal of the ADNI is to investigate whether the combination of neuroimaging, biological markers and clinical and neuropsychological assessments can accurately detect the disease progression in MCI and AD [68]. Ethical approval was obtained by the ADNI investigators (http://www.adni-info.org/pdfs/adni_protocol_9_19_08.pdf). All Institutional Review Boards of all participating sites at their respective institutions approved the study. All ADNI participants provided written informed consent before the start of the study.

### Participants

Subjects were selected in the present study according to the following criteria: Caucasian, availability of 3D T1-weighted MRI scan demographic information, MMSE and ADAS-Cog score. Full inclusion/exclusion criteria are described in detail at http://www.adni-info.org. Briefly, all subjects were between the ages of 55 and 90 years, were fluent in Spanish or English, and were free of any other significant neurologic diseases. LMCI participants had a subjective memory complaint, a Clinical Dementia Rating (CDR) of 0.5, and were classified as single- or multi- domain amnestic; EMCI group differed from LMCI only based on education-adjusted scores for the delayed paragraph recall subscore on the Wechsler Memory Scale–Revised Logical Memory II such that EMCI subjects were intermediate between normal subjects and LMCI. CN subjects had CDR scores of 0, and patients with AD met standard diagnostic criteria. According to our criteria, 192 participants, including 69 CN, 52 patients with EMCI, 41 patients with LMCI, and 30 patients with AD, were entered into the analysis.

### Vascular risk factor assessment

VRFs were evaluated at all in-person visits. This study focuses on six modifiable risk factors: diabetes (self-reported diabetes, use of antidiabetic therapy, or casual blood glucose≥200 mg/dl), hypertension (untreated systolic blood pressure≥140 mmHg, untreated diastolic blood pressure≥90 mmHg, or use of antihypertensive medications), smoking (self-report at least 20 cigarettes/day), depression (lifetime prevalence of major depressive disorder using the Diagnostic and Statistical Manual of Mental Disorders or International Classification of Diseases criteria), low education attainment (the proportion of adults with an International Standard Classification of Education15 level of 2 or less (pre-primary, primary, and lower secondary education)), and obesity (adult prevalence of body-mass index greater than 30 kg/m²).

To evaluate the summative effect of all VRFs, we used composite risk scores based on the summation of the individual relative risk value of each VRF [11]. Specifically, each of the indicated VRFs was assigned a 0 if absent or 1 if present based on the previously described criteria. The distinctive relative risk value of each VRF was then selected from the publication [11] and multiplied by the original value (0 or 1) to obtain the individual VRF scores. Finally, we summed these six VRFs to create a composite score for each participant.

### MRI data acquisition

High-resolution 3D T1-weighted MR images were acquired on a 3.0T GE scanner using the ADNI-1 (8-channel coil, TR = 650 ms, TE = min full, flip-angle = 8°, slice thickness = 1.2 mm, resolution = 256 × 256 mm and FOV = 26 cm) and ADNI-GO/ ADNI-2 (8-channel coil, TR = 400 ms, TE = min full, flip-angle = 11°, slice thickness = 1.2 mm, resolution = 256 × 256 mm and FOV = 26 cm). Both ADNI-1 and ADNI-GO/ ADNI-2 MRI data were acquired in the sagittal plane using an IR-FSPGR pulse sequence. Details about the ADNI MRI data acquisition protocol are available at the link http://adni.loni.usc.edu/methods/documents/mri-protocols/.

### Structural image analysis

Structural image analysis was carried out using VBM8 toolbox in Statistical Parametric Mapping (SPM8, http://www.fil.ion.ucl.ac.uk/spm/). The detailed procedure was as follows. First, all structural images were corrected for bias and segmented into gray matter (GM), white matter (WM) and cerebrospinal fluid (CSF) by a unified segmentation model [69]. The segmented images were then registered in brain templates by diffeomorphic anatomical registration using the exponential lie algebra (DARTEL) method [70]. Furthermore, the resulting images were normalized to the Montreal Neurological Institute (MNI) space. During the normalization step, the image intensity was modulated to preserve the original brain volumes. Finally, the modulated volumes were resampled to 2*2*2 mm^3^ and smoothed with an isotropic Gaussian kernel of 6 mm full width at half maximum (FWHM).

### Statistical analysis

### Demographic and neuropsychiatric characteristic analyses

One-way analysis of variance (ANOVA) and chi-square tests (only for the gender and APOEε4 gene frequency) were used to compare the demographic and cognitive performance data among the groups using SPSS 23.0 (https://www.ibm.com/support/home/). The statistical threshold was set at a p < 0.05.

### Group-level comparison of VRF scores and behavioral significance in the AD spectrum

One-way ANOVA was used to compare VRF scores differences in the disease spectrum. Partial correlation analysis was subsequently performed to test the relationship between the VRF scores and cognitive performance (MMSE and ADAS-Cog scores) in the AD spectrum (p < 0.05), after controlling for the covariates of age, gender, APOEε4 genotype, and total gray matter volume.

### Neural effects of VRF scores on GMV in the AD spectrum

Similarly, multivariate linear regression analysis was also performed to investigate the neural basis of VRFs on GMV in the AD spectrum (3dClustSim correction, p < 0.01, cluster size >1120 mm^3^):

$$\begin{array}{l} GM_{i}=\beta_{0}+\beta_{1}VRF+\beta_{2}Age+\beta_{3}Gen+\beta_{4}Edu\\ \;\;\;\;\;\;\;\;\;\;+\beta_{5}APOE+\xi \end{array}$$ (1)

where GM_i_ is the GM volume value of the *i*th voxel across group subjects and β_0_ is the intercept of straight line fitting in the model. β_1_ is the effect of the VRF scores on the GMV of the *i*th voxel. β_2_, β_3_, β_4_, and β_5_ are the effects of age, gender and APOEε4 genotype among the four groups, respectively, as covariates of no interest in the linear regression model.

In addition, multivariate linear regression analysis was used to analyze the potential effects of a single VRF on GMV in the AD spectrum.

### Group-level differences of GMV in the AD spectrum

Imaging data analysis was performed using the Analysis of Functional NeuroImages (AFNI) software (http://afni.nimh.nih.gov/afni). A voxel-wise analysis of covariance (ANCOVA) with age, sex and APOEε4 genotype as covariates of no interest was performed to determine the significant group differences of GMV among CN, EMCI, LMCI and AD. For multiple comparison correction, the latest version of the 3dClustSim program implemented in AFNI was used to control the false-positive rate (corrected p < 0.005, α=0.05, and cluster size=675 mm^3^). We subsequently extracted the GMV values of each region for a post-hoc analysis to determine the source of significance from the ANCOVA among the four groups.

### Relationship between cognitive performance and GMV in the AD spectrum

To investigate the neural substrates that underlie the functions of the MMSE and ADAS-Cog on the whole brain GMV, a multivariate linear regression analysis was employed (3dClustSim correction, p < 0.01, and cluster size >1120 mm^3^) [71, 72].

$$\begin{array}{l} GM_{i}=\beta_{0}+\beta_{1}MMSE+\beta_{2}Age+\beta_{3}Gen+\beta_{4}Edu\\ \;\;\;\;\;\;\;\;\;+\beta_{5}APOE+\xi \end{array}$$ (2)

$$\begin{array}{l} GM_{i}=\beta_{0}+\beta_{1}ADAS\text{-}Cog+\beta_{2}Age+\beta_{3}Gen\\ \;\;\;\;\;\;\;\;\;+\beta_{4}Edu+\beta_{5}APOE+\xi \end{array}$$ (3)

where GM_i_ is the GMV value of the *i*th voxel across group subjects and β_0_ is the intercept of straight line fitting in the model. β_1_ is the effect of MMSE or ADAS-Cog scores on the GMV of the *i*th voxel, respectively. β_2_, β_3_, β_4_, and β_5_ are the effects of age, gender and APOEε4 genotype among the four groups, as covariance of no interest in the linear regression model.

### Overlapping regions from the effects of VRFs on GMV and influence of GMV on cognitive performance in the AD spectrum

A conjunction analysis (the neural effects of VRFs on GMV overlapped with the neural substrates of the MMSE or ADAS-Cog on GMV) was then separately performed to identify the overlapping regions that were involved in the neural basis of VRFs and MMSE or VRFs and ADAS-cog.

### Mediation analysis

Given the significant association of VRF scores and cognitive performance on the severity of cortical atrophy observed in the AD spectrum, we performed a mediation analysis to determine whether cortical atrophy mediates the relationship between VRF scores and cognitive decline in the AD spectrum. The classic mediation model was selected, and the Sobel test was used to confirm the significance of the mediator if the weighted coefficient (a or b) was not significant [73, 74].

Three step regression models were constructed and are shown as follows:

Y=cX+e1 (4)

M=aX+e2 (5)

Y=c’X+bM+e3 (6)

where X is the independent variable (VRF), Y is the ndependent variable (cognitive performance), M is the mediator (cortical atrophy), a is the regression coefficient for the relationship between VRFs and cortical atrophy strength (GMV, same below) in regions of interest, b is the regression coefficient for the relationship between cortical atrophy strength in regions of interest and cognitive performance, c is the regression coefficient for the relationship between VRFs on cognitive performance, and c’ represents the effect of VRFs on cognitive performance while controlling for the indirect effect. We used the Bootstrap method to implement these steps [75]. The Bootstrapped iteration process with 10, 000 samples produced robust Bootstrapped Standard Errors (Boot SE) and 95% Confidence Intervals (CI) for the mediation effects. The significance of the indirect effects (a*b) was confirmed when 95% CIs were bound between (0 to +1) or (0 to −1) excluding zero.

## Supplementary Material

Supplementary Figures
