# Supplementary material for: Cortical atrophy mediates the accumulating effects of vascular risk factors on cognitive decline in the Alzheimer’s disease spectrum
Source: Aging (Albany NY). 2020 Jul 29;12(14):15058–76. doi: 10.18632/aging.103573 (PMC7425455; doi:10.18632/aging.103573)
Supplement: Supplementary Figures [file aging-12-103573-s001..pdf]

SUPPLEMENTARY FIGURES

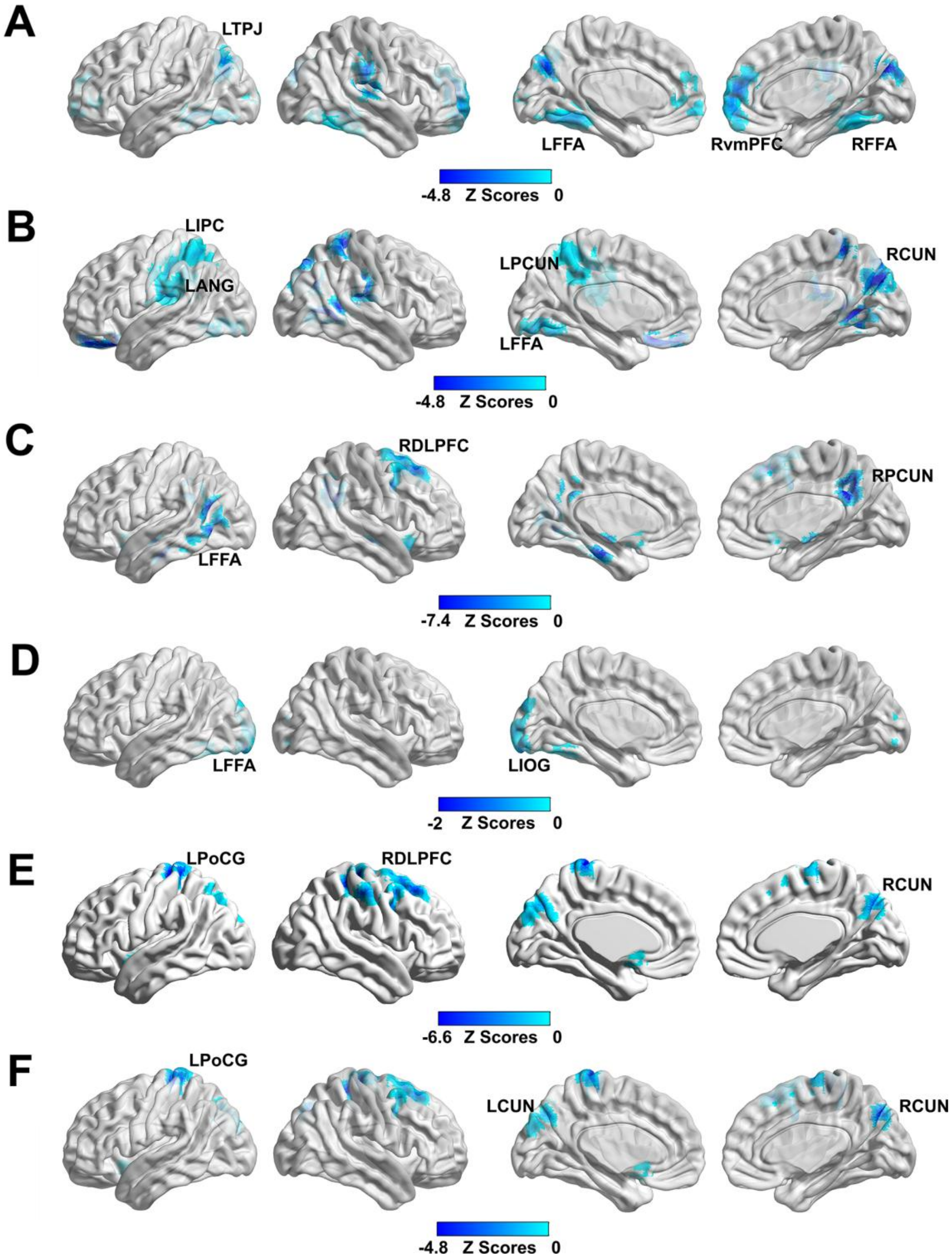

Supplementary Figure 1. Multivariate regression analysis revealed the significant effects of single vascular risk factor on gray matter volumes across all subjects (whole brain correction with sex, age and APOE ε4 genotype (AlphaSim correction, $p< 0.05$, Cluster size =3432mm^3)). Brain regions represented the significant effects of diabetes (**A**), hypertension (**B**), smoking (**C**), depression (**D**), obesity (**E**) and low education (**F**) on the GMV after controlling the effects of covariates including age, gender, APOEε4 genotype and group. Blue color indicates negative correlation between VRF scores and GMV in the AD spectrum. Color bar is presented with z scores. Abbreviation: LTPJ, left temporoparietal junction; LFFA, left fusiform face area; RvmPFC, right ventromedial prefrontal cortex; RFFA, right fusiform face area; LIPC, left inferior parietal cortex; LANG, left angular gyrus; LPCUN, left precuneus; RCUN, right cuneus; RDLPFC, right dorsolateral prefrontal cortex; RPCUN, right precuneus; LIOG, left inferior occipital gyrus; LPoCG, left postcentral gyrus; LCUN, left cuneus.

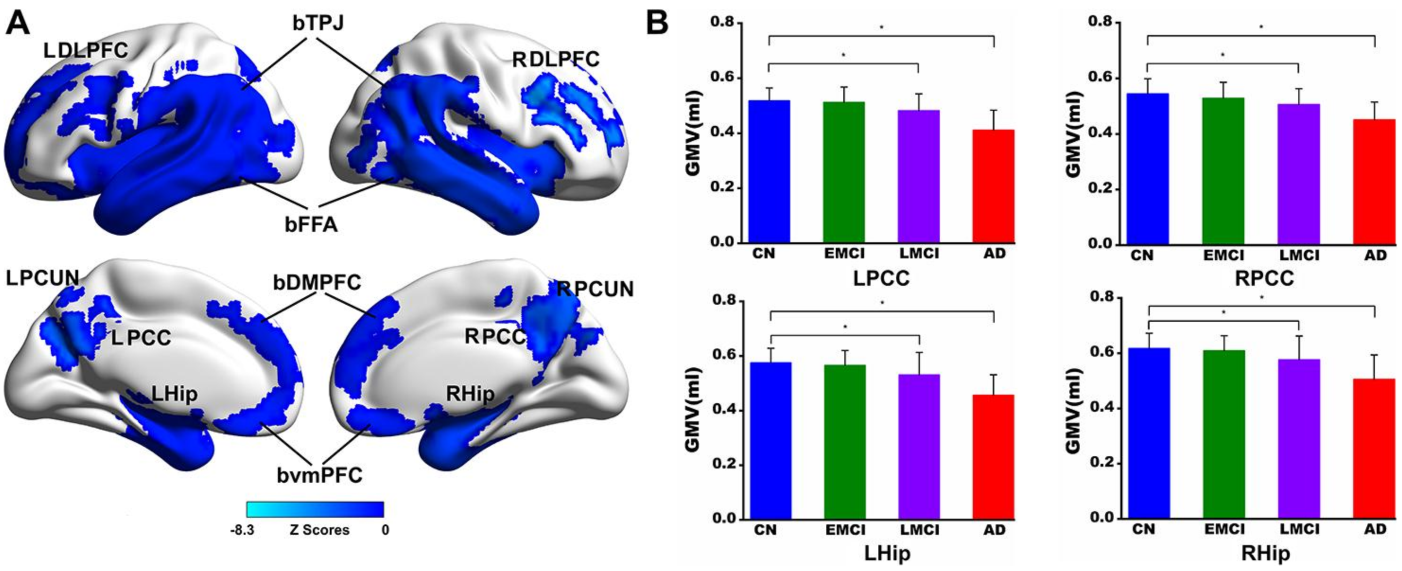

Supplementary Figure 2. Main effects of disease on the gray matter volumes across all subjects. (**A**) Brain regions with altered gray matter volumes were primarily located in the bTPJ, bFFA, bDMPFC, bvmPFC, LDLPFC, LPCUN, LPCC, LHip and RDLPFC, RPCUN, RPCC, RHip. Blue color indicates decreased gray matter volumes in the AD spectrum, color bar presents with z scores. (**B**) Numerical representation of the gradually decreased gray matter volumes in the target regions (LPCC: $F_{(3, 188)}$=27.61, p<0.000; RPCC: $F_{(3, 188)}$=19.98, p<0.000; LHip: $F_{(3, 188)}$=26.62, p<0.000 and RHip: $F_{(3, 188)}$=20.65, p<0.000) in the CN group (blue color), the EMCI group (green color), the LMCI group (purple color) and the AD group (red color). Abbreviation: bTPJ, bilateral temporoparietal junction; bFFA, bilateral fusiform face area; bDMPFC, bilateral dorsomedial prefrontal cortex; bvmPFC, bilateral ventromedial prefrontal cortex; RDLPFC, right dorsolateral prefrontal cortex; RPCC, right posterior cingulate cortex; RPCUN, right precuneus; RHip, right hippocampus; LHip, left hippocampus; LPCC, left posterior cingulate cortex; LPCUN, left precuneus; LDLPFC, left dorsolateral prefrontal cortex; CN, cognitively normal; EMCI, early mild cognitive impairment; LMCI, late mild cognitive impairment; AD, Alzheimer's disease; GMV, gray matter volume.

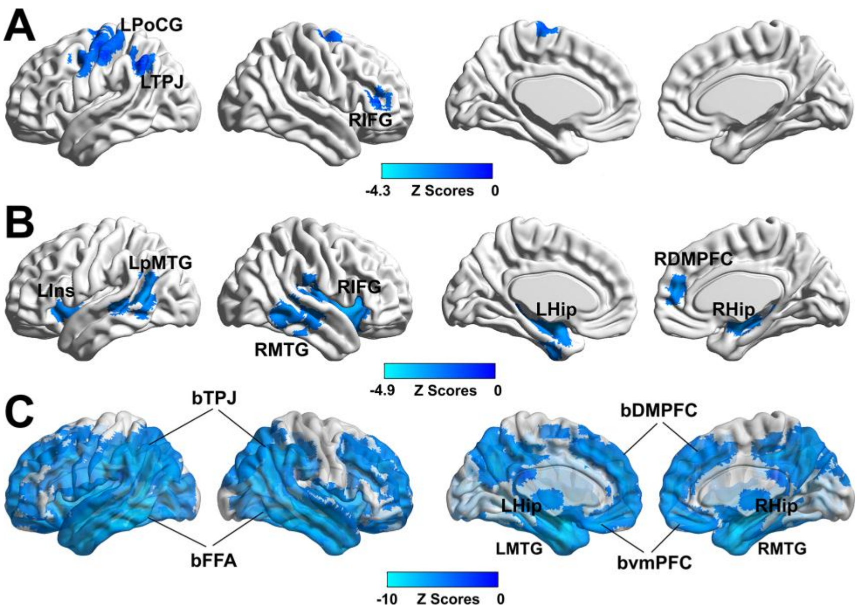

Supplementary Figure 3. Gray matter volumes were compared between CN group and EMCI group (**A**), CN group and LMCI group (**B**), and CN group and AD group (**C**). Blue color indicates decreased gray matter volumes in EMCI, LMCI and AD patients compared to CN subjects. Color bar presents with z scores. Abbreviation: LPoCG, left postcentral gyrus; LTPJ, left temporoparietal junction; RIFG, right frontal gyrus; LpMTG, left posterior middle temporal gyrus; LIns, left insular; RHip, right hippocampus; LHip, left hippocampus; RDMPFC, right dorsomedial prefrontal cortex; bTPJ, bilateral temporoparietal junction; bFFA, bilateral fusiform face area; bDMPFC, bilateral dorsomedial prefrontal cortex; bvmPFC, bilateral ventromedial prefrontal cortex; LMTG, left middle temporal gyrus; RMTG, right middle temporal gyrus; CN, cognitively normal; EMCI, early mild cognitive impairment; LMCI, late mild cognitive impairment; AD, Alzheimer's disease.

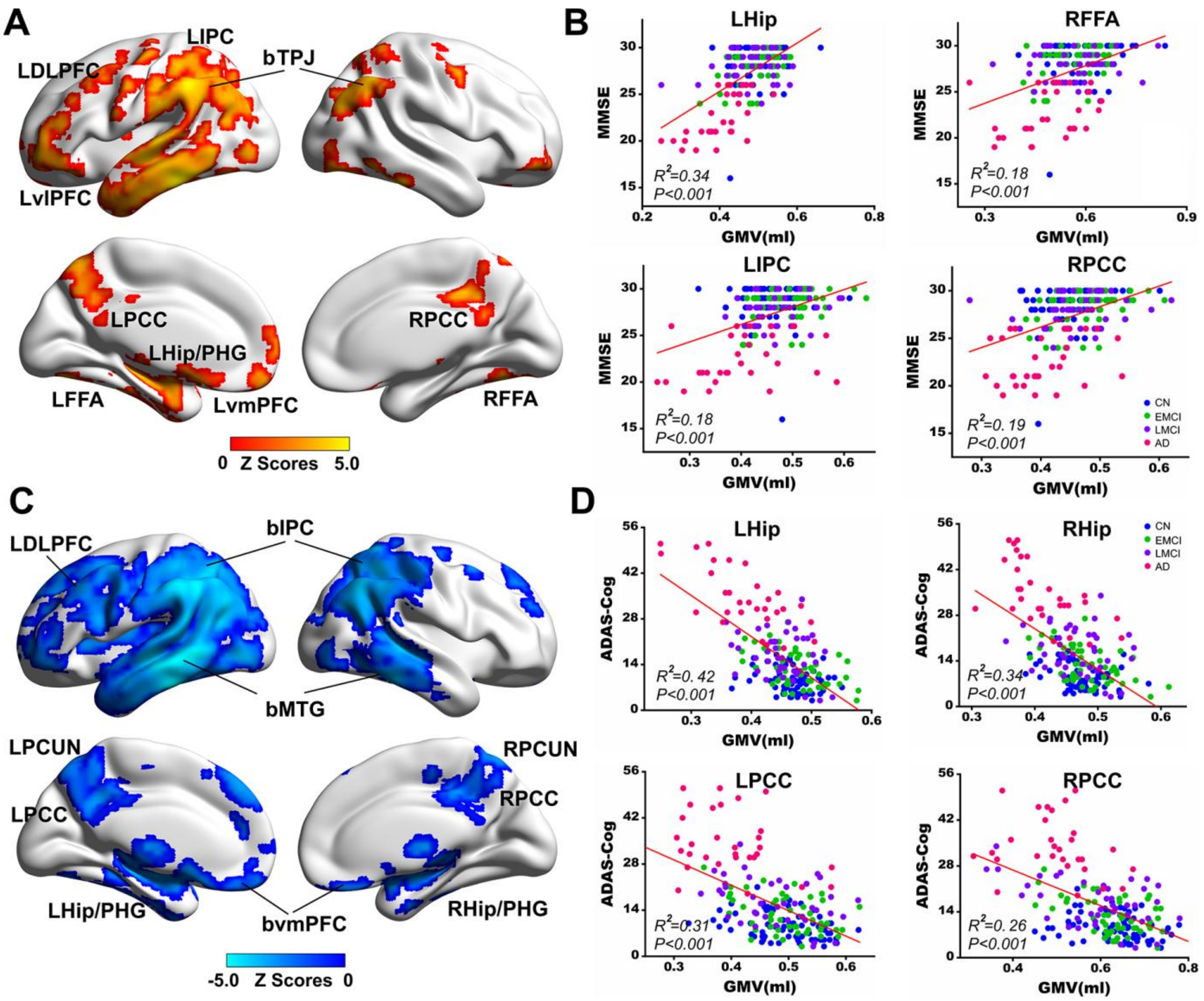

Supplementary Figure 4. Multivariate regression analysis revealed the significant effects of gray matter volumes on the MMSE and ADAS-Cog scores across all subject. (**A**) Brain regions represented the significant effects of gray matter volumes on the MMSE in the AD spectrum. Bright color indicates positive correlation between gray matter volumes and MMSE scores. Color bar presents with z scores. (**B**) Representative illustration of the relationship between gray matter volumes and MMSE scores. (**C**) Brain regions represented the significant effects of gray matter volumes on the ADAS-Cog scores in the AD spectrum. Blue color indicates negative correlation between gray matter volumes and ADAS-Cog scores. Color bar presents with z scores. D: Representative illustration of the relationship between gray matter volumes and ADAS-Cog scores. Abbreviation: LIPC, left inferior parietal cortex; LDLPFC, left dorsolateral prefrontal cortex; bTPJ, bilateral temporoparietal junction; LvlPFC, left ventrolateral prefrontal cortex; LPCC, left posterior cingulate cortex; RPCC, right posterior cingulate cortex; LHip/PHG, left hippocampus/ parahippocampal gyrus; LFFA, left fusiform face area; RFFA, right fusiform face area; LvmPFC, left ventromedial prefrontal cortex; bIPC, bilateral inferior parietal cortex; bMTG, bilateral medial temporal gyrus; bvmPFC, bilateral ventromedial prefrontal cortex; LPCUN, left precuneus; RPCUN, right precuneus; RHip/PHG, right hippocampus/ parahippocampal gyrus; MMSE, Mini-Mental State Examination; ADAS-Cog, Alzheimer's Disease Assessment Scale-Cognitive Subscale; CN, cognitively normal; EMCI, early mild cognitive impairment; LMCI, late mild cognitive impairment; AD, Alzheimer's disease; GMV, gray matter volume.

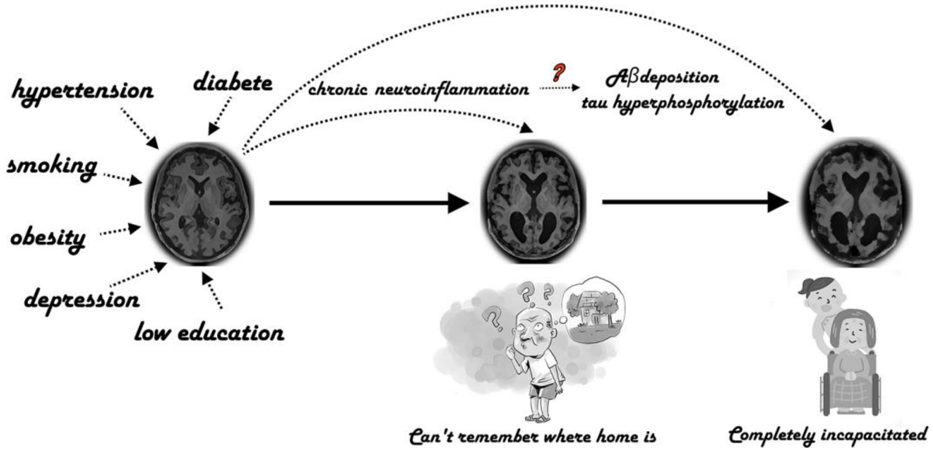

Supplementary Figure 5. Schematic of the proposed model on the mechanisms how VRFs affect cortical atrophy and cognitive decline in the AD spectrum. Single vascular risk factor act on the normal brain, leading to cognitive decline in patients. Vascular risk factors may cause chronic neuroinflammation, which in turn promotes Aβ deposition and hyperphosphorylation of the tau protein, ultimately leading to brain atrophy.
